# Lifestyle medicine for depression

**DOI:** 10.1186/1471-244X-14-107

**Published:** 2014-04-10

**Authors:** Jerome Sarris, Adrienne O’Neil, Carolyn E Coulson, Isaac Schweitzer, Michael Berk

**Affiliations:** 1 Department of Psychiatry, The University of Melbourne, 2 Salisbury Street, Richmond 3121 Victoria, Australia; 2 Centre for Human Psychopharmacology, Swinburne University of Technology, Melbourne, Australia; 3 School of Medicine, Deakin University, Geelong, Australia; 4 School of Public Health & Preventive Medicine, Monash University, Melbourne, Australia; 5 Florey Institute for Neuroscience and Mental Health, Parkville, Australia; 6 Orygen Youth Health Research Institute, Parkville, Australia

**Keywords:** Lifestyle, Depression, Exercise, Diet, Smoking, Alcohol, Prevention, Treatment

## Abstract

The prevalence of depression appears to have increased over the past three decades. While this may be an artefact of diagnostic practices, it is likely that there are factors about modernity that are contributing to this rise. There is now compelling evidence that a range of lifestyle factors are involved in the pathogenesis of depression. Many of these factors can potentially be modified, yet they receive little consideration in the contemporary treatment of depression, where medication and psychological intervention remain the first line treatments. “Lifestyle Medicine” provides a nexus between public health promotion and clinical treatments, involving the application of environmental, behavioural, and psychological principles to enhance physical and mental wellbeing. This may also provide opportunities for general health promotion and potential prevention of depression. In this paper we provide a narrative discussion of the major components of Lifestyle Medicine, consisting of the evidence-based adoption of physical activity or exercise, dietary modification, adequate relaxation/sleep and social interaction, use of mindfulness-based meditation techniques, and the reduction of recreational substances such as nicotine, drugs, and alcohol. We also discuss other potential lifestyle factors that have a more nascent evidence base, such as environmental issues (e.g. urbanisation, and exposure to air, water, noise, and chemical pollution), and the increasing human interface with technology. Clinical considerations are also outlined. While data supports that some of these individual elements are modifiers of overall mental health, and in many cases depression, rigorous research needs to address the long-term application of Lifestyle Medicine for depression prevention and management. Critically, studies exploring lifestyle modification involving multiple lifestyle elements are needed. While the judicious use of medication and psychological techniques are still advocated, due to the complexity of human illness/wellbeing, the emerging evidence encourages a more integrative approach for depression, and an acknowledgment that lifestyle modification should be a routine part of treatment and preventative efforts.

## Introduction

While modernity has provided multiple technological and medical advances including increased life-expectancy, it has come at a cost, in that a range of lifestyle issues are now negatively affecting our mental health [1]. As Hidaka [1] and Walsh [2] comment, in Western society people are increasingly becoming more sedentary and eating a poorer diet than previous generations. This, in combination with sleep/wake cycle pressures, substance misuse, and psychosocial factors such as more competition and time pressure, social isolation and less intimate engagement with the family unit, may exert a cost on mental health. Further, the combination of stress, fatigue, inactivity, and sleep deficiency in people who are “time-poor”, may advance obesity, and this in turn may promote a sedentary life with potential for resultant depression.

Due to the afore-mentioned challenges of modern urbanity, there is now the need to consider a “Lifestyle Medicine” approach for the potential prevention, promotion and management of depression. While medication and psychological interventions are first-line treatments for depression, Lifestyle Medicine offers a potentially safe and low-cost option for augmenting the management of the condition. While the evidence base remains patchy, many lifestyle or environmental factors are mutable and can provide the basis of practical interventions for the management of depression (summarised in Table 1). Lifestyle Medicine involves the application of environmental, behavioural, and psychological principles to enhance physical and mental wellbeing, adding a therapeutic and potentially preventative approach to illness [3]. This may involve modification of: diet; physical activity and exercise; relaxation and sleep-wake cycles; recreation and work-rest balance; and minimisation/avoidance of smoking, alcohol or illicit substances, in addition to the use of mindfulness-based meditation techniques [2]. Although the evidence base remains in its infancy, environmental issues are also considerations, such as reducing exposure to pollution (air, water, noise, and chemicals) and increasing time spent in nature, and are areas of current investigation. Activity scheduling such as encouraging engagement in meaningful activities and adequate social contact [1] is additionally of value. Further, Lifestyle Medicine may involve the application of clinical psychological techniques, insofar as motivational and behavioural factors are intrinsic to people trying to embrace lifestyle changes [3].

**Table 1:** Lifestyle medicine for depression

**Lifestyle element**	**Evidence level**	**Cost**	**Comment**
**Diet**	CS, LO	Moderate expense	Relationship found between dietary quality and depression; RCTs now required to validate
**PA/Exercise**	CS*, LO*, CTs	Inexpensive	Strong evidence of efficacy for improving mood
**Recreation**	OB, CTs	Variable expense	No studies exploring recreational activities for depression (aside from music therapy)
**Relaxation & meditative techniques**	CTs	Inexpensive	Evidence supports relaxation techniques (especially with a mindfulness component) in improving mood
**Sleep**	CS, LO, CTs	No expense	Strong causal link between sleep amount and quality, and depression risk
**Environment**	CS, LO, CTs	Potentially not adjustable	Association between reduction of pollution and mood; CTs showing NAT improves mood
**Socialization**	CS, LO	No expense	Strong association between social support/networks and mental health
**Animal/Pet therapy**	CS, CTs	Moderate expense	Studies support the psychological benefits of animals and pets
**Vices (smoking, alcohol)**	CS, LO	Potential to save money	Association between smoking and alcohol, and depressed mood

*CS* = Cross-sectional, *OB* = Observational Study, *LO* = Longitudinal, *CTs* - Clinical Trials, *NAT* = Nature-Assisted Therapy, *PA* = Physical Activity, Data assessing the relationship between exercise and depression has revealed mixed outcomes.

*Data assessing the relationship between exercise and depression has revealed mixed outcomes.

While lifestyle modification has been recognised by practitioners for centuries as a means by which to improve health outcomes, the field of “Lifestyle Medicine”, particularly in the context of mental health, is a relatively new field. While papers have discussed its broader application on health and in particular prevention of chronic disease and cardiovascular/metabolic conditions, little attention has been given to its application for mental health, and in particular depression, which is predicted to be the predominant cause of disability in the developed world [4], and is being argued as one of the prevalent non-communicable disorders [5]. Some studies show that patients with sub-threshold depression rate lifestyle or psychosocial approaches as strategies that are most helpful in improving their mood [6], while patients with clinical depression have rated exercise as the most effective intervention [7].

There is a heuristic theoretical framework explaining why the modern lifestyle may be impacting mental health. Obesity [8], poor diet [9], poor/decreased sleep [10], exposure to chemicals and pollutants [11], and high stress levels [12], may potentially disrupt the hypothalamic pituitary adrenal axis, increase cortisol and increase low-grade systemic inflammation and oxidative stress. Both neuroendocrine disruption and inflammation have been linked to the aetiology of depression [13,14]. Specifically, increased levels of proinflammatory cytokines, interferon gamma and neopterin, reactive oxygen and nitrogen species and damage by oxidative and nitrosative stress, in combination with lowered levels of antioxidants, may potentially damage mitochondria and mitochondrial DNA; this may result in neurodegeneration and reduced neurogenesis [14].

This opinion paper aims to provide a context for Lifestyle Medicine by providing an overview of the lifestyle factors that are linked with depression risk before exploring the evidence and clinical application of modifying these elements. The paper firstly explores data for which there is sound evidentiary support (diet, physical activity and exercise, mindfulness meditation, management of recreational substance misuse, sleep, and social interaction), and then touches on lifestyle and environmental elements that have nascent data and are subject to confirmatory investigation (greenspace and pollutant exposure, hobbies and relaxation, and animal/pet therapy).

## Review

### Lifestyle elements with supportive data

#### Diet

Major dietary changes have occurred across the globe in the past century [15]. Specifically, there has been a shift in the common dietary patterns of Western societies to typically include high levels of energy, saturated fats and refined sugar. In fact, nutrient-insufficient and energy-dense foods now contribute approximately 30% to the daily dietary intakes of American adults [16]. Over recent years, evidence has emerged to suggest that poor diet may be a risk factor for the onset of depression. While the strength and nature of this relationship remains unclear, not only have cross-sectional data shown that poor dietary patterns and quality are associated with depressed mood and anxiety [9,17-25], but prospective studies have further provided support for the predictive role of diet in the development of mental disorders.

Recent prospective data from the ongoing Whitehall II cohort study of middle-aged office workers in Britain (n = 3486) has revealed an increased risk of incident depression over five years for people consuming a Western style dietary pattern. Moreover, a reduced risk for those eating a whole foods diet pattern was observed [26]. Another recent prospective study, this time comprising Australian adolescents, has provided comparable data. An examination of examined 3040 Australian adolescents (aged 11–18) found that diet quality was associated with adolescent mental health both cross-sectionally and prospectively [27], where deterioration in diet quality was associated with poorer psychological functioning. Importantly, improvements in mental health were also mirrored by improvements in diet quality.

The notion that dietary modification can result in improved mental health outcomes is further supported by data from a large European study. The Spanish SUN Cohort study demonstrated an inverse association between the level of adherence to a Mediterranean dietary pattern and the risk for incident depression over approximately four years in more than 10,000 middle-aged professionals [28]. Importantly, these data did not support the reverse causality hypothesis -that depression causes poor dietary choices. Coupled with significant findings observed in other studies conducted around the world (Korea, Greece), these findings suggest a consistent pattern for the association between diet and mental health in various settings [29,30]. However, the issue of heterogeneity amongst studies is noted [31].

While the mechanisms underpinning the association between diet and mental health are not fully understood, various pathways have been proposed. Diet modulates several key biological processes that underscore mood disorders, including brain plasticity and function, the stress response system, mitochondria, inflammation, and oxidative processes [32,33]. A Western dietary pattern is associated with increased markers of systemic inflammation [34]. Further, an aetiological role for inflammation in the pathophysiology of depression is suggested by recent data showing that higher levels of serum high-sensitivity C-reactive protein (a marker of systemic inflammation) is an independent risk factor for a depressive episode [35]. Among people suffering from depression, systemic immune activation, characterised by increased levels of pro-inflammatory cytokines [36,37] and alterations in the acute phase protein response [38], have been observed. Another aspect of consideration, is that a wholefood diet provides protein and essential fatty acids, in addition to a range of micronutrients that are critical in neurochemical function, including B vitamins, zinc, magnesium, vitamin C, and a range of plant compounds such as flavonoids that are potent antioxidants and anti-inflammatories [39-41].

Altering dietary patterns may affect a variety of factors influencing the development and trajectory of depression [42]. While epidemiological data appear persuasive, currently no robust study has been conducted exploring the therapeutic impact of dietary changes on any diagnosed mental illness. Recent encouraging data suggests that dietary modification can reduce the risk of cardiovascular disease [34]. There are encouraging signs in a pilot randomised controlled study by McMillan et al. [43] that evaluated the effects of dietary change on mood and cognition in healthy individuals. The study examined 10 days of adherence to a nutrient-rich diet, compared to no dietary change in 25 young female adults. The dietary change group showed significant improvements in self-rated vigour, alertness and contentment compared to the control group. Other studies comprising depressed populations have demonstrated the benefits of dietary modification using supplements such as omega 3 polyunsaturated fatty acid (PUFAs) on mood outcomes. Recent meta-analyses [44-46] have highlighted the benefits of PUFAs (in particular eicosapentaenoic acid) in those with depression; specifically, for those with greater symptom severity. However, high heterogeneity of the studies was observed.

While these emerging data provide potential validity for the role of dietary and nutritional factors in the genesis of depression and its management, it should be acknowledged that currently, there is minimal evidence for dietary modulation as a treatment for depression. A current clinical trial [47] is however underway to test this hypothesis by assessing the effect of a prescriptive diet versus an inactive control (befriending). The results of the RCT are due in 2015. Regardless of the current deficit of empirical evidence for prescriptive dietary advice to treat depression, it is recognized that diet has a major impact on comorbid medical conditions that are exceedingly more common in people diagnosed with depression, including cardiovascular disease and metabolic disorders [48], and the precautionary principle should guide practice.

#### Physical activity and exercise

Modernity has similarly reduced the amount of work- and leisure-time physical activity (PA) and formalized exercise undertaken by the average person. Our lifestyles are increasingly sedentary, with the resultant side-effect of obesity currently recognized as a major health problem worldwide [49,50]. Epidemiological studies have shown that adequate PA (based on clinical guidelines) is associated with fewer depressive symptoms, while insufficient PA may be a risk factor for the development of depressive symptoms [51-54]. This risk factor may be best modified in early development, [55] as regular PA in childhood is associated with reduced risk of developing depression in adulthood. This effect was found even after adjusting for adult PA. Pasco et al. [56] also showed a similar protective effect of PA in the elderly. Not all studies have found such as association: a longitudinal analysis (1991–2002) of the effects of exercise on depressive symptoms among 5952 genetically identical twins (aged 18–50) showed that an increase in exercise participation did not predict fewer depressive symptoms [57].

Aside from general PA having a potential relationship with mental health, formalised exercise appears to be an effective mood elevator [58-60]. Exercise is also a relatively cheap and safe intervention that has been shown to provide a range of additional health benefits [61]. Aside from exercise providing marked beneficial effects on neuroendocrine systems, it also increases self-efficacy and self-esteem (via activity scheduling and attainment of goals) which are important psychological issues among people who are depressed [62]. Furthermore, exercise may have additional beneficial effects by increasing social engagement and enhancing body image.

Exercise has been shown to moderate a range of biological pathways including: inflammatory cytokines, oxidative stress, neurotrophins and neurogenesis [32,63]. In animal models, exercise has demonstrated biological effects via multiple mechanisms, including increased levels of brain derived neurotropic factor (BDNF) [64,65], and enhanced neurogenesis [66]. Modulation of monoamine systems has also been shown, with PA increasing expression of 5HT in animal models [67], and this is theorised to explain some of the antidepressant effect of exercise [60]. The long-term chronic effects of regular exercise may assist in regulating the neuroendocrine axis and produce a normalisation of cortisol levels [68], while increasing circulating beta–endorphins [69] (for a review see Moylan *et al*. 2013) [70].

Evidence provides some support for the use of exercise. A recent Cochrane review (updated from 2009) included 32 studies (n = 1858) involving exercise for the treatment of researcher-defined depression [71]. From these studies, 28 RCTs (n = 1101) were included in a meta-analysis revealing a moderate to large effect in favour of exercise over standard treatment or control. However, only four trials (n = 326) with adequate allocation concealment, blinding and ITT analysis were located, resulting in a more modest effect size in favour of exercise. Pooled data from seven trials (n = 373) with long-term follow-up data also found a small clinical effect in favour of exercise.

Adequate PA and exercise is of synergistic relevance given the increased burden of obesity and the metabolic syndrome in individuals with psychiatric disorders [48], and the well-established benefits of PA in those comorbid health conditions. Currently, the balance of evidence supports the use of exercise of adequate intensity and duration to improve mood and reduce depressive symptoms and comorbid medical disorders.

Another form of exercise that combines a “mindfulness” element (discussed further below), is the practice of yoga. A recent systematic review located 12 RCTs (pooled n = 619) [72]. For effect on the severity of depression, there was moderate evidence showing a beneficial short-term effect of yoga compared to usual care, and limited evidence compared to relaxation, and aerobic exercise. Aside from the physiological benefits from yoga, the mindfulness component may also provide a form of focus that may quell rumination (which is common in depression). It should be noted that while yoga is gaining increased popularity in the Western world, its safety needs to be considered. While Yoga is regarded as generally safe, a previous review has revealed 76 cases of adverse events from its practice [73]. Due to this, yoga should be practiced carefully under the guidance of a qualified instructor.

#### Mindfulness meditation

Meditative practices may also have an application in improving mood and preventing the tumescence of a depressive episode. While the concept of meditation is varied, it can be categorized into “open monitoring” or “focused” forms. A key aspect of meditative practice involves mindfulness, commonly defined as the awareness which arises through “paying attention in a particular way: on purpose, in the present moment, and non-judgmentally” [74]. The use of meditation as a Western behavioral intervention was pioneered by Kabat-Zinn [75], with the formal training program known as Mindfulness-Based Stress Reduction (MBSR). The practice of MBSR is centered on an 8–10 week structured program which involves (a) training in mindfulness meditation practice, (b) mindful awareness e.g. during yoga postures, and (c) mindfulness during stressful everyday situations and social interaction. Meditative practices such as MBSR can be readily incorporated into people’s lives, and requires only basic training (although more advanced traditional techniques, or modern techniques involving CBT, can also be taught).

Regular meditation has been shown in various neuroimaging studies to affect a range of biological changes such as, alterations in gray matter morphology, increased cortical thickness in the prefrontal cortex (PFC) and right anterior insula, increased oxygenated haemoglobin in the anterior PFC, and elevations in whole blood serotonin (5-HT) levels [76,77]. Electroencephalographic (EEG) studies have revealed a significant increase in alpha and theta activity during meditation [78]. Evidence supporting mindfulness is growing, with a review and meta-analysis of the intervention for anxiety and mood reduction revealing a large effect size on improving mood symptoms for participants receiving mindfulness-based training [79]. It should be noted that the quality of most studies are poor, and it is often difficult to ascertain which form/s of meditation is more appropriate for use in depression.

#### Management of recreational substances (alcohol, cigarettes, caffeine)

Epidemiological studies and community surveys indicate that alcohol abuse and dependence commonly co-occur with affective disorders. Although the specific mechanisms of the associations are still being elucidated [80], acute alcohol use increases release of monoamines [81]. While acute alcohol use reduces neuronal glutamate release, during alcohol withdrawal (colloquially known as the “hangover” stage) a greater amount of rebound glutamate is released from the synapses and in combination with dysregulated monoamine and neuroendocrine pathways, may provoke anxiety and dysphoria [81]. A meta-analysis of four large-scale epidemiological studies found a two- to three fold increased lifetime risk for both depressive and anxiety disorders in those with alcohol dependence or abuse [80]. This has been further supported by a more recent meta-analysis which found that the presence of either an alcohol use disorder or major depressive disorder doubles the risk of the other disorder [82]. There is also data from prospective community studies that shows heavy use of alcohol in adolescence and young adulthood predicts later onset of major depressive disorder [83]. Further, harmful or hazardous alcohol use (defined by ICD-10 criteria) when compared with light drinkers in early adulthood predicts greater depressive symptomatology, even after controlling for confounding factors [84]. Several studies have shown that depressed mood is substantially alleviated within a very short time after abstaining from alcohol [85-87]. A study of 235 women with postpartum depression who were consuming above recommended levels of alcohol, found a brief alcohol intervention (two clinician-guided sessions providing safe drinking guidance and homework tasks), to be effective at reducing depressive symptoms (compared with no treatment intervention) at six-months following the intervention [88].

Smoking cigarettes increases the risk for the genesis of affective and anxiety disorders [89-93] and appears to be a potential risk factor for the development of de-novo depression [94]. Cigarette smoking in adolescence may also increase the risk of the later development of clinically significant depressive symptoms [95]. There is a risk of escalation of smoking in people with depression [91], with some evidence for the existence of a shared genetic vulnerability to both smoking and depression [96]. In both unipolar depression and bipolar disorder (but perhaps not schizophrenia), smoking has not only a deleterious effect on symptom severity [97], but may also interfere with response to treatment [98,99]. A greater risk of continued smoking and lower abstinence rates may also be associated with sub-threshold depressive symptoms [100]. Smoking for some may be a form of self-medication for acute dysphoric symptom reduction [91,101]. Regular smokers can be viewed as being in a persistent dysphoric withdrawal state, punctuated by brief intoxications when ingesting nicotine. A key neurobiological mechanism linking smoking and mood involves the dopaminergic system, which is implicated in addiction. Aside from involvement in the regulation of mood, dopamine has a crucial role in the reward/addiction pathways [102]. As mentioned above, depression can be seen as having an inflammatory component, and smoking also aggravates inflammation and provokes marked oxidative stress [103]. Additionally, evidence from a cross-sectional study indicates that second-hand cigarette smoke is positively associated with depressive symptoms in smoking-naive people (even after adjustment for age, race/ethnicity, gender, education, alcohol consumption, and medical comorbidities) [104].

Caffeine is in many cultures the most commonly used psychoactive substance. It acutely increases attention, alertness, cognition and mood, with some individuals with dysphoric mood being predisposed to use caffeine more heavily, due to its mood-elevating potential (via activation of noradrenergic and dopaminergic pathways) [105]. Caffeine modulates the adenosine system, and the anxiogenic potential of caffeine is influenced by polymorphisms of the A2A receptor [106]. There is a suggestion of dysregulation of the adenosine system in depression [107]. While evidence supports the avoidance of caffeine in anxiety disorders [108], data suggests that caffeine use from coffee consumption may be protective against depression. A prospective longitudinal analysis (1996–2006) of 50,739 US women (mean age, 63 years) who were free of depressive symptoms at baseline, found that there was a reduced risk of diagnosed depression at follow up for those consuming two to three cups per day compared to those consuming one or less per week [109]. This effect was even slightly stronger in those consuming over four cups per day. Interestingly, decaffeinated coffee was not associated with depression risk. Regardless, there is the potential for people with high caffeine use affecting insomnia, and this in turn may increase risk or aggravation of depressed mood [110]. High caffeine containing energy drinks are increasingly popular, and there is a suggestion, albeit methodologically preliminary, of an association of use with mood disorders [111-113].

#### Sleep

A good night’s sleep is critical for mental and physical health, and affective disorders are associated with major disturbance in circadian rhythm [114,115]. Sleep disturbance is a frequent symptom of depression, and a strong causal link exists between insomnia and depression [116]. Further, residual sleep difficulties may be predictive of subsequent depression relapse. It is likely that this is bidirectional; that is, insomnia can increase depression risk and vice versa. Research has shown that people with chronic insomnia have an increased risk of a major depressive disorder. Cross-sectional data of 2,619 individuals from the Netherlands Study of Depression and Anxiety (NESDA) revealed that people with a current depressive or anxiety disorder, or remittent depression had a significant association with sleep disturbance. Interestingly, sociodemographic factors and psychotropic medication use was not found to contribute to sleep outcomes [117]. A review of 21 prospective studies with follow-up data by Baglioni [118] found that insomnia predicted two-fold increased risk for a depressive episode. Epidemiological data have shown that approximately 40% of chronic insomniacs suffer from another comorbid psychiatric disorder [10]. Aside from poor sleep affecting mood, data from the Behavioral Risk Factor Surveillance System of 138,201 individuals found that sleep disturbance is a significant risk factor for myocardial infarction, stroke, obesity, diabetes, and coronary artery disease [119].

Current treatment strategies are mainly based on sleep hygiene techniques and cognitive behavioural techniques; and via the use of hypnotic or antidepressant medications [120]. Additionally, the use of lifestyle modification programs may improve sleep by addressing factors associated poor sleep e.g. sedentary life, poor diet, caffeine and alcohol use. Merrill and colleagues [121] analyzed data from a lifestyle change program involving 2,624 individuals aged 30 to 80 years from the Illinois USA area. Participants were assessed at baseline and then again after four weeks of completing a 40 hour program which educated them on making long-term improvements in nutrition and physical activity and sleep habits. While the study was uncontrolled and the assessments were potentially open to bias (thus caution in interpreting this study is advised), results revealed that of the 10% of participants who were experiencing insomnia, 64% no longer had a sleep disturbance at the conclusion of the study. Increased PA and a lowering of BMI and caffeine consumption was associated with improved sleep. Changes in alcohol consumption and smoking however were not found to significantly contribute to improvement in sleep.

#### Social interaction

Another key lifestyle influence affecting mental health concerns a person’s social environment. Positive, supportive, intimate relationships, be it via family, friends or a relationship, have been established to have a beneficial effect on general health, and in particular for maintaining psychological health [122]. Ibarra-Rovillard and Kuiper [122] propose that the extent to which social relationship partners are perceived to fulfill (or undermine) basic psychological needs serves to explain both the positive and negative effects on the well-being of depressed individuals. Data from the English Longitudinal Study of Aging, observed whether baseline positive and negative exchanges with partners, family and friends were linked to two-year changes in depression [123]. Results showed that after adjusting for confounding factors, negative, but not positive, exchanges with family and friends were associated with greater occurrence of depression. A positive relationship has also been shown between social support and leisure time physical activity (LTPA) [124]. A prospective cohort study of 5395 middle-aged adults participating in the British Whitehall II study found that amongst participants who reported recommended levels of LTPA at baseline, adults who experienced high emotional support were more likely to report recommended levels of LTPA at follow-up. These findings suggest that the enhancement of social networks and increased social support may help the individual to maintain the recommended level of LTPA. While adequate LTPA may potentially provide a psychologically beneficial effect, data is needed assessing its relation to depression.

### Lifestyle elements with emerging data

#### Recreation and relaxation

A vital lifestyle element in the management of depression is the pursuit of balancing the “work-rest-play” dynamic. While the specific effect of pleasurable recreational activities as a treatment for clinical depression per se has not been adequately evaluated, it is plausible that this is a meaningful consideration. Recreational activities provide an opportunity to experience pleasure, to direct the mind away from rumination and worry, and may provide a setting for increased social interaction. The benefits of participation in organised physical recreation have been investigated as one strategy for enhancing mental health and wellbeing. As Street, James and Cutt comment [125], evaluations by government departments in Australia and the US have found that people who participate in organised recreational activity enjoy better mental health, are more resilient against the stresses of modern living, with a reduction of depressed mood also evident; however the direction of the association remains speculative. One common form of inexpensive recreation involves listening to music. Studies of varying methodological quality have been conducted assessing this for reducing depressive symptoms [126]. A systematic review of 17 studies found that listening to music over a period of time helps to reduce depressive symptoms in adults. No specific type of music was found to be superior, with personal preference recommended.

Aside from recreational activities that may provide psychological relaxation, formalized relaxation techniques may also be incorporated into people’s lifestyle. A Cochrane Review and meta-analysis by Jorm and colleagues [127] of 11 trials using relaxation techniques (progressive muscle relaxation, relaxation imagery, autogenic training) versus control (wait-list, no or minimal treatment), found that although clinician-rated outcomes were non-significant, self-rated depressive symptoms were reduced, with a moderate effect size reported. Specifically, a meta-analysis of five trials found that relaxation reduced self-reported depression compared to wait-list, no treatment, or minimal treatment post intervention (although these are not optimal control conditions). It should also be noted a meta-analysis of nine trials revealed that relaxation produced less effect than psychological interventions on self-reported depression.

#### Environmental factors

A range of transpersonal elements are modifiable factors that may have effects on mental health and depression. External factors that may affect health can be categorized into *environmental*: nature/greenspace; climate, season, pollution (noise, air and water quality, chemical exposure) and *social*: interaction with family, friends and relationships, and a therapeutic association with animals or pets. It should be noted that while there is evidence of social interaction and support being potentially protective in depression (discussed above), there is currently only weak evidence regarding the other elements.

Adequate exposure to nature (greenspace) may provide theoretical benefits for general health. Although being close to greenspace may not necessarily be correlated with increased PA, studies have shown a relationship to a range of positive health effects [128]. The benefits of spending time in nature may also involve increased exposure to fresh air and sunlight. While the evidence of specific health benefits from “nature-assisted” therapy (for example nature hikes) is unclear due to a deficit of rigorous studies, the extant evidence suggests a therapeutic effect. A systematic review by Annerstedt and Wahrborg [129] found that for studies of moderate to low evidence grade, health improvements were reported in 26 out of 29 studies. Exercising in nature also may synergistically increase wellbeing beyond PA in an urban setting [130].

It is a commonly held belief that people feel better “when the sun is shining”, however whilst acute exposure to sunlight and a pleasant temperature may anecdotally enhance mood, there appears to be an inconsistent relationship between seasonal variations and the prevalence of depression [131,132]. The beneficial effect of sunlight exposure on mental health has been suggested to be mediated, in part, by vitamin D [133]. However recent research involving 198 people with multiple sclerosis who were followed prospectively for an average of 2.3 years, showed that greater exposure to sunshine may decrease depressive symptoms, despite no correlation to vitamin D levels [134]. Low levels of vitamin D appear to be associated with depression risk, although there is conflicting evidence as to the effects of supplementation on improving mood [33,133,135]. Currently, the breadth of evidence provides inconsistent support for the use of vitamin D supplementation for depression.

Other environmental factors that are under current investigation pertain to noise pollution, and exposure to technological devices. While no direct link to depression has been documented, continual hyperstimulation is a theoretical concern for both cognition and mental health. Environmental toxins (including particulate air pollution) are also an issue, having a potential effect on the central nervous system [136]. Emerging evidence suggests that air pollution may induce neuroinflammation, oxidative stress, microglial activation, cerebrovascular dysfunction, while potentially altering the blood–brain barrier [136]. A recent novel mouse model study investigated whether long-term (10 months) exposure to ambient fine airborne particulate matter compared to filtered air, affects affective and cognitive responses [137]. Results revealed that mice exposed to long-term air pollution displayed more depressive-like responses and impairments in spatial learning and memory compared with mice exposed to filtered air. Exposure to air pollution has been found to be associated with an increase in depressive symptoms in a longitudinal study of 537 elderly Koreans [11]. However, the evidence currently does not clearly confirm the relationship between pollution and depression, with NHANES data (unpublished) failing to demonstrate a consistent relationship between persistent organic and heavy metal pollutants and depression.

#### Animal and pet therapy

Humans commonly have close relationships with animals, particularly pets, and such relationships may have a theoretical benefit for alleviating depressed mood. Having a pet can provide physical affection and a feeling of unconditional love, assist in the maintenance of a routine, and also provide responsibility and an additional sense of life purpose. Formalized animal-assisted therapy may involve horses (equine therapy), dogs, or even interactions with mammals such as dolphins. Time spent with farm animals by people with psychiatric disorders may reduce depression and state anxiety, and increase a sense of self-efficacy [138], with a small and methodologically limited study suggesting beneficial effects of pet therapy on mood and perceived quality of life in 21 elderly inpatients affected by dementia, depression and psychosis [139]. No clinical recommendations are possible given the available data.

### Clinical considerations

The application of Lifestyle Medicine should be considered in the context of long-term sustainability. Motivational issues, time restrictions, financial limitations, the perspective about the source of their difficulties and treatment priorities, may influence a patient’s ability to implement lifestyle changes. Adherence and engagement are increased by having ownership of a treatment plan and a sense of shared partnership in its development and planning. Treatment strategies should therefore be developed taking cognisance of the above factors, and the treatment package should be individually tailored and offered in a step-wise manner. It is also necessary to consider readiness to change when engaging in discussion regarding lifestyle modification, and concordant with the transtheoretical model, to only embark on this when the person has moved beyond precontemplation to the contemplation or preparation stages [140]. Some lifestyle choices and “vices” may provide the person self-perceived support and comfort, and in such cases change needs to be handled delicately. Often, strict advice promoting abstinence, or a demanding diet or exercise regime, may cause added suffering, and may also provoke guilt if the person cannot meet these expectations, or lead to paradoxical behaviour if oppositional cognitive schema are activated.

With respect to specific dietary advice, it is important to recognise that the nutrients critical for neurological function (magnesium, folate, zinc and essential fatty acids), and are all components of a healthy diet, found primarily in foods such as leafy green vegetables, legumes, wholegrains, lean red meat and fish. Foods rich in polyphenols (e. g. berries, tea, dark chocolate, wine and certain herbs), are also valuable for cognitive and cardiovascular function [141,142]. Aside from having adequate wholefoods, people are also advised to reduce processed foods, refined carbohydrates and sugars, and transaturated fats. Adherence to the Mediterranean diet has also been shown to be associated with a range of mental health benefits.

Regarding the dosage and type of exercise to recommend, research indicates that a dose-dependent effect occurs, with regular moderate to strong intensity exercise eliciting more positive results [143,144]. Clinical guidelines for exercise recommend physician assessment (or referral to an exercise physiologist) before commencing a new regime, which should consist of moderate to vigorous aerobic exercise (30–60 minutes) in addition to anaerobic weight-bearing exercises approximately four to six days per week [145,146]. Exposure to social interaction and nature when exercising may be useful on theoretical grounds.

The integration of meditative practices can be readily incorporated into most people’s lifestyle. While regular formal practice of meditation or yoga can be recommended, it can be applied in simple forms, such as mindful walking or eating, or yoga breathing exercises. With respect to yoga, it is advised that people pursue instruction under a qualified instructor, and proceed in a graded manner; as is the case for all forms of exercise. Patients with glaucoma (with any inverted poses), osteoporosis (with any heavy weight-bearing pose), should proceed with caution.

Due to the potential impact of heavy alcohol use on the risk and treatment for depression [147,148], routine clinical questioning about alcohol consumption is appropriate. In a clinical setting, the focus should be on differentiating and managing problem drinking (where it is identified), and on psycho-education of patients regarding modest alcohol consumption and avoiding heavy episodic drinking. Further education may be required regarding potential interactions between alcohol use and medications. Techniques combining motivational interviewing and cognitive behavioural therapy may be of benefit in treating the dual components of depression and problem drinking [149,150].

Smoking cessation should also form a routine component of clinical care, with individuals being offered evidence-based smoking cessation interventions when appropriate [151]. Interestingly, PA used concurrently with smoking cessation seems to be protective against relapse in quitters, further reinforcing recommendations regarding exercise [152]. One caveat is required: the act of cessation is associated with withdrawal symptoms including transient dysphoria, irritability and a risk of aggravation of depression [153]. However, once the neurochemical set-point has re-adapted to the absence of nicotine, this should normalize. While trials of smoking cessation that examine long term change in depression risk have not been conducted, recent data suggests that quitting smoking is associated with better social functioning and self-perceived health status [154].

Sleep hygiene techniques that can be offered in the case of poor sleep or insomnia, includes focusing on adjusting caffeine use, limiting exposure to the bed (sleep restriction) with the patient having only a limited time to sleep, and getting up at a set time in the morning [155]. This should regulate the circadian rhythm, which is of particular significance in people with affective disorders [115]. Reducing exposure to light prior to sleep to increase melatonin secretion is of theoretical value [156], as is increased exposure to morning sunlight upon waking. Further sleep hygiene advice includes stimulus control: avoidance of stimulating activity and stimulants close to sleep (for example, smoking, caffeine and stimulating TV or books), and quiescent sleep preparation. Although evening meal portions should not be excessive (this may cause rebound hypoglycaemia), sufficient calorific intake is required to avoid waking up due to hunger.

Other lifestyle targets can consist of emphasising the importance of social contact, and the theoretically positive benefits of animals/pets [122,139]. The therapeutic benefits of spending time in nature is supported by a limited dataset [129]. Adequate exposure to sunlight should enhance vitamin D levels and serotonin turnover [157], however this needs to be balanced with concerns over skin cancer, thus time of day/year and skin colour are factors in consideration about the amount of sun exposure recommended. While current data are weak with respect to an association with depression, limited exposure to environmental toxins, chemicals pollutants [136] and noise pollution [158], is also a valid general health consideration. While limited study in this area has been pursued and there is no direct link to depression, moderation of excessive technological interface (e.g. mobile phones, computers, television) is also a potential consideration in Lifestyle Medicine [2].

### Summary

While many factors, including genetics, personality and cognition, and environmental stressors contribute to the aetiology of depression, lifestyle components may have an important role in the disorder’s pathogenesis. Currently, the contemporary clinical management of depression has had more of a focus on medication and psychotherapy, and to date, has not widely incorporated the evidence regarding many lifestyle factors into guidelines. As outlined above, there is evolving evidence to support the modification of selected lifestyle elements. Further research is now needed to study “integrative models” assessing the application of prescriptive Lifestyle Medicine, similar to those used in other medical populations [159]. Trial designs could explore individualized tailored programs, or the step-wise application of individual components according to patient interest or need. While the study would not be double-blinded, it is possible to randomize participants into the active lifestyle modification group versus a placebo or inactive group, with the outcomes being assessed by an independent blinded researcher (using validated psychiatric scales). Further work exploring the mechanistic underpinnings of lifestyle modification is also of benefit.

In future respect to the research underpinning lifestyle modification for depression, aside from prospective RCTs needing to assess some lesser studied lifestyle elements such as dietary modification, one critical area of study is to explore the effects of an integrated multi-faceted application of lifestyle medicine. To date, to our knowledge only one RCT has examined the effects of combining multiple lifestyle adjustments for the treatment of depression. Garcia-Toro and colleagues [160] conducted an RCT of 80 outpatients with diagnosed major depressive disorder (non-SAD) who were taking antidepressant treatment. Four specific lifestyle recommendations consisting of dietary modification, exercise, sunlight exposure and sleep patterns were prescribed in the active group, while the control group was given instruction to perform a pattern of eating, sleeping, exercise and exposure to light which they considered might make them feel better. Blinded assessment was conducted before and after the six month intervention period. The active group had a significantly greater reduction of depression than the control group, with 11 out of 40 people (28%) in the active group achieving remission (HAM-D < 7), compared to only one person in the control group. These encouraging results are in line with remission rates of standard antidepressants, and support the conduct of similar more definitive studies.

## Conclusions

In conclusion, a variety of lifestyle modifications exist that have appropriate potential front-line clinical application alongside pharmacotherapies and psychological techniques to better manage depression. While the judicious use of medication and psychological techniques are still advocated, due to the complexity of human illness/wellbeing, the time has come for a more integrative approach for depression, and an acknowledgment of the potential applicability of lifestyle modification.

## Competing interest

No authors note any direct competing interest.

## Authors’ contribution

All authors contributed content and editing to the manuscript. All authors read and approved the final manuscript.

## Pre-publication history

The pre-publication history for this paper can be accessed here:

http://www.biomedcentral.com/1471-244X/14/107/prepub
